# Policy Series: The Role of a Stakeholder Member Group in Shaping Geriatric Policy: The National Association for Geriatric Education

**DOI:** 10.1093/geroni/igaa057.1799

**Published:** 2020-12-16

**Authors:** Leland Waters, Brian Lindberg

## Abstract

The National Association for Geriatric Education (NAGE) is a non-profit membership organization representing Geriatric Workforce Enhancement Programs (GWEPs), Geriatric Academic Career Awardees (GACAs) and other programs that provide education and training to health professionals in the areas of geriatrics and gerontology. Our work includes faculty training and fellowships, continuing education, and hands on experiences in the clinical setting. One of our priorities is to educate policy makers and the public about the need for health care professionals to receive geriatrics education so they will better serve the expanding older population. One of our goals is to provide a mechanism for policy development and dissemination to external audiences regarding the mission, goals and impact of geriatric education programs. Our policy objectives include providing guidance to the United States Public Health Service and other organizations in the development of programs to enhance the education of health care practitioners and others. Another objective is to educate Congress about necessary priorities in geriatric education. We serve as a voice for the goals and interests of the nation’s GWEPs, GACAs, and other groups providing education in geriatrics and gerontology. This symposium will first describe how geriatric educators inform policy. Then a historical perspective of how NAGE has influenced aging policy is provided. Recent efforts to increase funding for geriatric education will be shared, followed by future directions in policymaking.

